# Designing and delivering facilitated storytelling interventions for chronic disease self-management: a scoping review

**DOI:** 10.1186/s12913-016-1474-7

**Published:** 2016-07-11

**Authors:** Enza Gucciardi, Nicole Jean-Pierre, Grace Karam, Souraya Sidani

**Affiliations:** Ryerson University, School of Nutrition, 350 Victoria Street, Kerr Hall South, room 349-I, Toronto, ON M5B 2 K3 Canada; North York Family Health Team, 240 Duncan Mill Rd, Suite 707, Toronto, ON M3B 3S6 Canada; Humber River Family Health Team, 245 Dixon Road, Etobicoke, ON M9P 2 M4 Canada; Ryerson University, Daphne Cockwell School of Nursing, 350 Victoria Street, Toronto, ON M5B 2 K3 Canada

**Keywords:** Self-management, Chronic illness, Storytelling, Narration, Peer support, Narrative, Group education, Chronic disease, Chronic illness, Patient education, Patient self-management, Health education, Self-care

## Abstract

**Background:**

Little is known about how to develop and deliver storytelling as an intervention to support those managing chronic illnesses. This scoping review aims to describe the core elements of storytelling interventions in order to help facilitate its implementation.

**Methods:**

A scoping review was conducted in seven databases for articles published up to May 2014 to identify interventions that describe in detail how storytelling was used to support people in disease self-management interventions.

**Results:**

Ten articles met all inclusion criteria. Core elements consistently observed across the storytelling interventions were: reflection and interactive meaning-making of experiences; principles of informality and spontaneity; non-directional and non-hierarchical facilitation; development of group norms and conduct to create a community among participants; and both an individual and collective role for participants. Differences were also observed across interventions, such as: the conceptual frameworks that directed the design of the intervention; the type and training of facilitators; intervention duration; and how session topics were selected and stories delivered. Furthermore, evaluation of the intervention and outcome assessment varied greatly across studies.

**Conclusion:**

The use of storytelling can be a novel intervention to enhance chronic disease self-management. The core elements identified in the review inform the development of the intervention to be more patient-centred by guiding participants to take ownership of and lead the intervention, which differs significantly from traditional support groups. Storytelling has the potential to provide patients with a more active role in their health care by identifying their specific needs as well as gaps in knowledge and skills, while allowing them to form strong bonds with peers who share similar disease-related experiences. However, measures of impact differed across interventions given the variation in chronic conditions. Our findings can guide future development and implementations of storytelling interventions.

## Background

Self-management is imperative when living with a chronic illness. In order for those living with such diseases to be effective managers of their condition, they need to gain useful knowledge and practical skills, be motivated to change their lifestyle, and develop coping strategies to overcome barriers and negative attitudes [1]. Various interventions are available to assist people in effectively managing their illness. However, results of studies that evaluated these interventions specifically for self-management education and support indicate a high attrition rate, low adherence rate, and low satisfaction with the content / topics covered, as persons exposed to these interventions found the content not quite relevant and congruent with their specific needs that are often related to day-to-day management [2–5]. This state of affairs led to exploring alternative interventions to improve upon how we deliver self-management education, care and support for those living with a chronic condition. Storytelling has emerged as a potential new approach to support disease self-management.

Telling stories is a natural and universal form of communication [6]. Stories are value laden, creative, and representative of a person’s experiences and understanding of their world [7]. The narration of one’s story can give an individual power over how they wish to be represented [8] and it fosters self-reflection [9]. Storytelling is the communication of these reflections using sounds, images or words, with improvisation frequently included [7]. Simply put storytelling, whether done in writing or orally, is the sharing of a personal narrative. Storytelling is a term synonymous with story-sharing, discussion circles, group circles, and Talking Circles.

Within the healthcare context, storytelling is emerging as a means of assistance in learning about and managing one’s disease [10]. The use of storytelling within disease management is premised on the fact that each person has his or her own unique experiences living with and managing a disease; thus peoples’ accounts are a valuable information source to both themselves and others. Listening to the stories can assist patients to reflect on their own experiences and recognize others may be experiencing similar struggles and circumstances, thereby enabling pertinent information to be disseminated on how to deal with / manage their condition. By allowing for open dialogue in a safe and caring environment there is an opportunity for patients to be actively engaged [10] in the shared stories. By identifying with the storyteller, participants can become invested in the content and be positively influenced by the self-management actions described. For instance, storytelling may break down cognitive resistance to messages promoting lifestyle and behavioural changes [10], thus creating a setting conducive to patients’ increased receptivity to the health information in the stories. This may motivate them to incorporate new behaviours into their lives [11]. Within the health care context, this reduction in change resistance could potentially lead to positive health outcomes [10] as the patient is more inclined to follow self-management strategies that have reportedly worked for others [11] that they may have previously avoided. There is a mutual benefit to storytelling when participants exchange their health-related stories, which can potentially result in the discovery and exploration of new information, practical management strategies and skills, and related resources [12, 13], providing opportunities for adoption of resolutions [14] to ongoing management issues.

When provided in a group format, storytelling allows for discussion of the complexities and practicality of disease management and of the unique needs of individuals over time. Storytelling can establish a network of trust and equality among participants, build cohesion among participants [15], reduce stigma associated with disease, and develop relationships amongst the participants [16]. This may be particularly meaningful for those who would normally find it difficult to express themselves to others without the disease [16]. Storytelling can also create an opportunity for organizational learning. Organizational learning is a collective process whereby a group of individuals learn and understand different issues through open dialogue [9]. For example, the traditional technique of Talking Circles used by American Indian tribes is a customary tool that has served many purposes including education on tribe traditions and cultures, health education, and health promotion [17]. Talking Circles is expected to create a comfortable and safe environment for participants to share knowledge, ask questions, and relate to one another [17]. Experienced group members are especially valuable resources for organizational learning as they can offer insight into how to deal with the unique challenges that disease management poses over time. As a result, storytelling can naturally facilitate peer support and enable a support network to form [18]. Peer support through storytelling may encourage individuals to examine their emotions, problem-solving skills, goal setting and exchange social support, all of which are vital self-management components [19]. Lastly, storytelling fits well with the patient-centered paradigm as it focuses on the patient’s perception of their unique needs and their ability to self-manage their disease. This approach can facilitate patients to develop strategies to manage their illness [20]. Therefore storytelling can be a relevant and potentially effective tool to disease management in the current healthcare system.

Although storytelling presents as an exciting approach for chronic disease self-management, relevant literature is scarce. Specifically, information on the conceptual underpinning, development, delivery, structure, and outcome assessment of storytelling interventions for chronic disease self-management is not well-established or defined. The purpose of this scoping review is to better understand the core elements and the principles guiding the design and implementation of storytelling interventions within the context of chronic disease self-management.

## Methods

### Search strategy and screening

A scoping review is commonly used to ‘map key concepts underpinning a research area and the main sources and types of evidence available’ [21, 22]. This type of review was conducted to examine the core elements that characterize storytelling as a chronic disease management intervention and to identify gaps in the existing literature [21, 22]. We searched the following databases: Ovid-Medline, Web of Science, HealthSTAR, CINAHL, ProQuest Nursing & Allied Health Source, PsycInfo, and Cochrane Library, for all articles up to May 2014. In consultation with a librarian, the key search terms were specified as: *narration, storytelling, story-sharing or anecdote; group-experience; group circle or talking circle; chronic disease or chronic illness; self-management or self-care; and intervention studies, evaluation, health education or programs*. Articles were limited to scholarly publications and academic journals.

A total of 607 articles were found: 260 from Ovid-Medline, 109 from Web of Science, 64 from HealthSTAR, 55 from CINAHL, 46 from ProQuest Nursing & Allied Health Source, 43 from PsycInfo, 28 from Cochrane Library, and 2 from hand searching *Oncology Nursing Forum* journal. Titles, abstracts, and full-texts were sequentially screened independently by two researchers to determine eligibility. We retrieved the full papers of citations which passed the initial screening, and two reviewers independently assessed each against the eligibility criteria. Reviewers compared results and resolved any discrepancies through discussion with a third party. We also used forward and backward citation searching techniques to identify further literature; however no additional articles were found using this method. To further identify grey literature, authors of the articles included in the review were contacted to obtain their training and intervention manuals.

### Inclusion and exclusion criteria

**Fig. 1:**
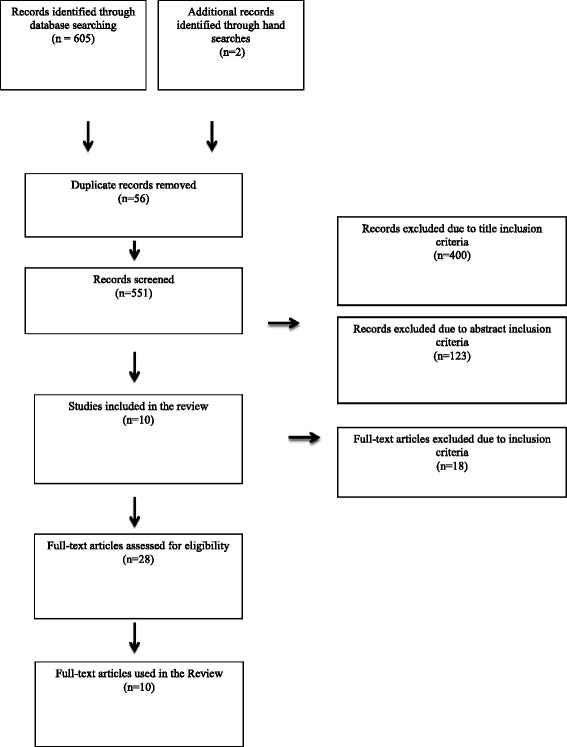
Selection process of studies based on search strategy

Articles were included if they met the following criteria: (1) the main intervention under evaluation consisted of a storytelling or narration approach; (2) a description of how the intervention was developed and delivered was provided or could be obtained by contacting the author(s); (3) the intervention focused on self-management aimed to improve the mental, physical, or psychosocial health of patients with a chronic disease (whether physical such as cancer or diabetes, or psychiatric such as depression); and (4) the intervention involved the sharing and discussing of stories (oral or written) among at least two participants. Articles were excluded if: (1) they were not written in English; (2) the intervention was a ‘narrative-based therapy’; and (3) storytelling occurred solely between health professional(s) and a patient. In total, ten articles were included in our review (see Fig. 1).

### Data extraction & analysis

One reviewer independently extracted data from each article using a standard attribute and process form developed for this review. For each article, information on the objectives, publication year, intervention location, type of chronic illness experienced by participants, study design, and sample size were noted. Also noted were the participant demographic characteristics, facilitator type and duties, conceptual framework underlying the intervention design, structure of intervention delivery (e.g. how were stories told/shared and reflected upon), length and duration of intervention, session topics, identified story themes, measurement tools, method of study, data analysis, and author suggestions and limitations. A second reviewer independently examined and verified data extraction to confirm accuracy and relevance. Intervention features across studies were then independently compared and contrasted by one reviewer who organized the information according to similarities and differences. Discussions regarding the collected data was held among all authors until mutual agreement could be reached on the emerging results. Ethics approval was not necessary for this research as it did not involve human subjects and it is solely a review of the existing literature.

## Results

### Description of studies

Two sets of articles reported results of the same study that evaluated the same intervention in the same population [12, 15, 23, 24], and the remaining articles described independent interventions [17, 20, 25–28]. In total, the articles addressed eight interventions. Four papers were from the United States [17, 23–25], three from the United Kingdom [12, 15, 26], two from Europe [20], Netherlands [27] and one from Australia [28]. The interventions were primarily conducted with adults, with one intervention conducted with an adolescent population. Target populations predominantly had diabetes or cancer from various age groups and cultural backgrounds. One study used quantitative [24], seven used qualitative [15, 17, 20, 23, 25, 27, 28], while two used mixed [12, 26] methodologies for data collection and analysis. Four of these were pilot projects [12, 23–25]. The study designs utilized were randomized controlled trials [12, 15, 23], randomized pre/post-test control group [24, 25], observational closed cohort [27], participatory action research [26, 28], phenomenology [17], and narrative qualitative [20]. Additional characteristics of the included articles can be found in Tables 1 and 2.

**Table 1:** Characteristics of studies ᅟ

Author, year, Country	Study design, sample size	Health condition	Participant age, gender	Ethnicity or race, Socioeconomic Status	Study objective	Conceptual framework for intervention	Facilitator
Comellas (2010), [25] United States	Pilot Study using pretest/post-test controls, *n* = 17	Diabetes Mellitus	66.8 years (average), 71 % female	Minority Adults in Urban communities	To improve diabetes self-management behaviors by becoming more physically active, eating healthier, adhering to medication, solving problem and setting goals.	Not Stated	Community Health Promoters
Crogan, Evans & Bendel (2008)^a^, [24] United States	Descriptive pilot project using pretest/post-test controls, *n* = 7	Cancer	48–74 years, 86 % female	Unknown	To evaluate symptom reports and the impact of a nurse-led storytelling intervention occurring in a supportive group setting	Watson’s (1985) 10 Carative Factors	Nurse
Evans, Crogan & Bendel (2008)^a^, [38] United States	Descriptive single blind pilot project, *n* = 10	Cancer	48–74 years, 86 % female	Unknown	To develop a nurse-led storytelling intervention for oncology patients, and implement the intervention using trained oncology nurses	Watson’s (1988) Theory of Human Caring	Oncology nurse educators
Greenhalgh et al. (2011a)^b^[12], United Kingdom	Pilot randomized controlled trial, *n* = 79 (10–12 per group)	Diabetes Mellitus	Unknown	Minority ethnic, Low income	To refine and test the new complex intervention in diabetes education; informal story-sharing group	Not Stated	Bilingual Health Advocate
Greenhalgh, Collard & Begum (2005b), [26] United Kingdom	Action research framework drawing on thematic and narrative analysis *n* = 42	Diabetes Mellitus	Unknown	Multi-ethnic, Low income	To develop and refine complex interventions for diabetes support and education in minority ethnic groups	Not Stated	Bilingual Health Advocate
Greenhalgh et al. (2011b), [15] United Kingdom	T hematic and narrative analysis *n* = 82 (groups of 7–12)	Diabetes Mellitus	25–82 years, 73 % female	African Caribbean & Bangladeshi & Tamil & Punjabi/Urdu & Somali, Low income	To analyze narratives of people with diabetes to inform design of culturally congruent self-management education programmes	Not Applicable	Bilingual Health Advocate
Koch & Kralik (2001), [28] Australia	Participatory Stringer’s Action Research Approach *n* = 8	Multiple Sclerosis & Urinary Incontinence	52 years (average), 100 % female	Unknown, Mixed income	To describe the development and implementation of an action research program focusing on understanding the experience of living with chronic illness	Not Stated	1st author (a nurse) in 1st group, inexperienced research student in 2nd group
Piana (2010), [20] Italy	*N* = 94 (total) Descriptive narrative	Diabetes Mellitus	16 years (average), 44 % female	No socio-demographic data were considered.	To induce a narrative-autobiographical approach in the care and education of adolescents with type-1 diabetes and observe the effects of this novel approach on adolescents’ self-awareness, concern for self-care, and well-being.	Narrative-Autobiographical Approach	Doctors, Nurses, Educators, Trainers, Dieticians, Psychologists
Sitvast (2013) [27], the Netherlands	Multiple-case design, *n* = 42	Psychiatric Disorders	Unknown	Unknown	To investigate whether the process of making photo stories in health care matches with requirements of self-motivation in self-management programs	Social Cognitive & Ecological Theories on Health Behavior	Nurses and Occupational Therapists
Struthers et al. (2003) [17], United States	Descriptive phenomenological, *n* = 147 (5–20 per circle)	Diabetes Mellitus	Unknown	Native American, Unknown	To find out what the experiences of American Indian Talking Circle participants are	Not stated	Community members with expertise in the culture

(^a^ or ^b^) same intervention

**Table 2:** ᅟDescription of interventions and outcomes

Author, Year, Country	Session number, frequency and duration	Session topic examples	Tools, props, action orientation	Outcomes	Measurement tools	Method of data analysis
Comellas (2010) [25], United States	5 sessions	Diagnosis, self-management, goal setting, sexual health	Goal setting	Physical and nutrition self-care activities and overall well-being.	Surveys (SDSCA measure), World Health Organization 5-item Well-Being Scale,	Comparisons were made from baseline data to evaluate change from pre to post intervention
Crogan, Evans & Bendel (2008)^a^, [24] United States	12 weekly sessions, 1.5 h long	Diagnosis, living with disease, loss of control, relationships, death	N/A	Pain	McGill Pain Questionnaire	Repeated measures analysis of variance
				Stress	Index of Clinical Stress, Cantril’s Ladder	
				Self-efficacy	Physical Self-Efficacy Scale	
				Mood	Satisfaction with Life Scale, Brief Depression Rating Scale	
				Coping	Index of Clinical Stress, Cantril’s Ladder	
				Satisfaction with Life	Satisfaction with Life Scale, Brief Depression Rating Scale	
Evans, Crogan & Bendel (2008)^a^, [37] United States	12 weekly sessions, 1.5 h long	Coping, control issues, life, hope, desires, fear, relationships	N/A	Healing for clients and their relationships; finding meaning in & transforming suffering; acceptance of life journey, including death	Index of Clinical Stress	Exit Interviews, Facilitator debriefing questionnaires
					Cantril’s Ladder	
					McGill Pain Questionnaire	
					Satisfaction With Life Scale	
					Brief Depression Rating Scale	
				Other qualitative data	Exit Interview	
				Ability of the nurse facilitator to effectively implement storytelling techniques and differentiate storytelling group from the control group	Facilitator Debriefing Questionnaire	
Greenhalgh et al. (2011a)^b^, [12] United Kingdom	72 biweekly sessions, 2 h long	Feeding the family, medication, dealing with doctors	Pills, food samples	Primary outcome (a composite of blood pressure, smoking status, lipid ratio, atrial fibrillation, and HbA1c)	UKPDS (UK Prospective Diabetes Study) coronary risk score	Statistical comparison
				Secondary outcomes included attendance	Observation	
				Secondary outcomes included HbA1c	Blood test	
				Secondary outcomes included well-being	Psychometric questionnaire	
				Secondary outcomes included confidence in managing and living with illness	Patient Enablement Instrument (PEI)	
Greenhalgh, Collard & Begum (2005b), [26] United Kingdom	Unknown	Diagnosis, diet, exercise, check-ups, medications, shopping, feelings	Pills, insulin, glucose meters, letters, activities (eg. self-monitoring, cooking, trying exercises, looking at shoes)	Mean Glucose Concentration	Blood test	Constant comparative method
Greenhalgh et al. (2011b), [15] United Kingdom	13 biweekly sessions, 2 h long	Diagnosis, weight loss, diet, exercise, medication	Food samples, glucose meters, artifacts (eg. hospital letters, tablets), exercising, group trips	Stories told How stories inform program design	Ritchie & Spencer’s ‘framework’ method Narrative analysis Interpretive analysis	Ritchie & Spencer ‘Framework’ (2003), Narrative analysis and Interpretive analysis using Bakhtin’s (1981) dialogical approach and Riessman’s (2008) notion of storytelling as performance
Koch & Kralik (2001), [28] Australia	10 sessions (40 h of contact)	Sex, incontinence, life with disease	Creating, implementing, and evaluating plans of action	Cycles of look, think, act in PAR approach	Observation	By research team concurrently with data generation
Piana (2010), [20] Italy	9 days (2 h autobiographical approach, 1.5 h diabetes self-management education)	Diagnosis, challenges of living with diabetes, relationship with food, relationship with one’s own body, with others and self care.	Writing, communication through songs, poems, readings, images, drawings and creative workshops	Stress reduction, change in self-perception, perception of relationships with others and with the disease itself	Questionnaires with open ended questions	Qualitative analysis on the open-ended questions
Sitvast (2013) [27], the Netherlands	8 weekly sessions	Family, friends, pets, hobbies, independence, jobs	Photos, goal setting and planning activities	Moral Learning Self-Motivation Action	Framework of methodological steps	Structural analysis on a meta level grounded in the tradition of interpretivism and ethnography
Struthers et al. (2003) [17], United States	12 sessions	Diabetes (perceptions, facts, prevention), nutrition (basics, preparation traditional foods), healthy lifestyles (physical, emotional, family, community)	Flip charts, visual aids, symbolic item (eg. feather or rock)	Individual anthropometrics Participant experience	Pretest (introductory session) & post-test (final session) for individual anthropometrics Clinic health charts also reviewed, Interviews	Comparative, Phenomenological, Verification from participants

(^a^ or ^b^) same intervention

### Designing storytelling interventions

#### Conceptual frameworks

Using a conceptual framework to develop a health intervention provides insight on the active ingredients and mechanisms responsible for the expected changes in outcomes. There is no common literature to suggest a specific conceptual framework for storytelling or a storytelling intervention for chronic disease self-management. A few articles identified specific conceptual frameworks: social cognitive and ecological theories of health behaviour [27], the nursing philosophy of caring and healing [23, 24] and narrative-autobiographical [20].

#### The core principles of storytelling interventions

The most prominent objective across the interventions was to get participants to reflect on their illness experience and create meaning from it through storytelling [15, 20, 23–25, 27, 28]. At the core of all the interventions’ agendas was the importance of participants finding personal meaning from self-reflection, as well as from the shared meaning-making processes which occurred within the group context when discussing the various interpretations of the stories. Stories also facilitated significant meaning in the context of relationships with family, friends, and work colleagues [23, 24], and they comprised actual life events and reflections or were representative of opinions and emotions.

Several principle components were common in the design of the interventions reviewed. First, informal and spontaneous sharing of stories occurred at each session [12, 15, 17, 20, 23, 25–28]. Second, health professionals (e.g. dietitian, nurse, and/or doctor) used a non-directive facilitation approach [12, 15, 23, 25–28] without the didactic delivery of information. Facilitators, however, would respond to the group’s shared stories [12, 15] and all provided information when asked. The only exception occurred in one study where directive facilitation was permitted and built into the intervention in order to provide necessary disease-specific health information [17]. Third, the facilitator was considered as an equal to the participants [17, 23–25, 28]. Lastly, community norms were established amongst the group members grounded by trust, respect, empathy and no judgment [12, 15, 17, 23, 24, 26–28].

#### Facilitators and additional contributors

Four of the interventions were facilitated by healthcare professionals with experience in both adult education and disease management [20, 23, 24, 27, 28]. The remaining four interventions accessed community members with varying education levels and teaching experience as peer facilitators, who were familiar with the participants’ culture and language(s) spoken [12, 15, 17, 25, 26]. In one study facilitators needed to be bilingual while also having the disease or living with a family member with the disease, which allowed the facilitators to be familiar with the participants’ conditions and enabling them to relate to the patients on a more personal level [25]. In three studies conducted by the same research team, the community member facilitators were called Bilingual Health Advocates [12, 15, 26]. In another study, facilitators were titled Community Health Promoters [25]. Regardless of who the facilitators were, their primary role consisted of drawing out personal stories, encouraging discussion of these stories, supporting group processes, and encouraging the sharing of disease management tips between group members. In one intervention, the facilitators were active participants in the storytelling process as a way of role-modelling the notion of trust [23]. In other interventions, guest healthcare providers or consultants (e.g. podiatrist) were called upon for their expertise on a specific session topic [12, 15, 26, 28].

#### Facilitator training

Seven of the ten articles discussed training for facilitators [12, 17, 20, 23–26]. The training consisted of a one-time session lasting eight hours for nurse facilitators [23, 24], a 12-week course of three-hour sessions that included a trainers’ workbook for Bilingual Health Advocates [12, 14], or a 5 week course of two hour sessions for Community Health Promoters. Training for the health professionals was conducted to provide insight and instruction on how to create a safe, caring and non-judgmental environment, as well as how to manage group conflict [23]. Training also provided instructions on the components of a Talking Circle [17] or a traditional storytelling format [12, 26], how to explain the concept of storytelling to a group of participants [12, 26], and to facilitate the sessions in a non-didactic manner [12]. During training, health professionals discussed and practiced storytelling principles and guidelines [23, 24]. Some intervention training, particularly for community member facilitators, included additional education for specific diseases [12, 17, 26]. The Bilingual Health Advocates benefitted from extensive training which addressed their specific learning needs to improve overall confidence in group facilitation [12, 26].

### Delivering storytelling interventions

#### Session number, duration and attendance

The intervention sessions were given over 5 to 13 weeks, except in one study [12]. The length of each session varied, lasting one hour [20], one-and-a-half [23, 24] or two hours [12, 15] (Table 2). In contrast, Greenhalgh et al. delivered 72 sessions lasting two hours over a 6 month intervention period [12]. The number of participants in a session ranged from 3 [23] to 20 [17], and one intervention was delivered in a camp setting to 38 participants [20].

#### Atmosphere of sessions

The intervention sessions were designed to create an open atmosphere in which everyone had the opportunity to speak about their personal experiences and to reflect on the meaning of the stories. In order to create such a setting, group norms, rules, and community values were collaboratively established during the first several sessions [12, 15, 17, 23, 24, 26–28]. These actions were also acknowledged in order to set the tone for the program in regard to acceptable conduct and participant behaviour. The seating arrangement during the sessions was described in three studies as a circle [15, 17] or as selected by participants [26].

#### The nature of stories told

Session topics were primarily selected in advance by participants [12, 15, 26, 28]. Selecting topics in advance meant participants could come to each session prepared with the stories they intended to share on the topic. Alternatively, the topics could be chosen by group participants at the commencement of each session [23, 24, 27]. In the initial session participants tended to discuss their diagnosis experiences as an introductory topic [15, 24, 26]. Across all interventions stories focused on issues which participants were most concerned about or self-management areas they needed help with (Table 2). In two interventions participants created realistic and attainable self-management goals [25, 27].

#### How stories were shared

One of the interventions was unique in that it provided “social time” for participants to begin sharing stories prior to the actual storytelling sessions [12, 15]. During this social time, participants formed smaller groups labelled as “buzz groups” [12]. Once regrouped to commence the storytelling session, participants could bring up any issues raised during the buzz groups to gain the benefit of a larger group discussion of those topics [12]. In the initial portion of sessions for six interventions facilitators guided the sharing of personal stories in an unstructured, spontaneous, and informal way. Often issues from the previous sessions were revisited at the start of a subsequent session [23, 28] indicating more than one topic could be discussed in a single session. This also demonstrated more time may be needed for additional reflection on a specific topic to further stimulate learning. Participants often shared stories, and the significance of these stories was discussed by the group [23, 24, 27, 28]. Their accounts were primarily dispersed verbally [15, 17, 23, 24, 28], but at times action-oriented activities were also included (e.g., cooking, exercising, demonstrating blood glucose testing) [26] along with shared pictures and props [27]. In the camp intervention, participants were required to anonymously write and share their feelings and thoughts regarding their disease by using songs, poems, and readings as communication tools to express themselves [20]. Using props stimulated further action among group members; for example, participants purposefully compared their sugar levels using glucose meters during class [26] and passed around samples of rice as a prop when discussing the glycemic index [12].

In five interventions, once stories were revealed, discussions of lifestyle as they relate to mental, emotional, and physical aspects of self-management, ensued [15, 17, 25, 26]. For some interventions, an educational component and disease-specific education materials were provided based on the session topic [15, 17, 26]. Periods of silence were also found to be valuable; the investigators [23, 28] noted the importance of silent moments for contemplation and reflection on stories. After each participant had contributed to the discussion, the events of the day were summarized by the facilitator and the session was concluded [17]. As reported in two studies [15, 17], stories and discussions often lingered on even after the session ended and some participants traveled home together.

### Collective and individual role of participants

All articles described their interventions as participant-centered; participants had substantial control over the programs’ agenda and delivery. Regardless of when it occurred, participants were encouraged to self-reflect on their personal disease experience, consider the stories shared within the group, and participate in group discussions [15, 27, 28]. Over time, participants naturally took on the role of sharing and listening to stories, discussing and providing feedback to one another [23, 24]. They also provided progress updates on their self-management goals [15, 27]. Self-reflection was intended to move towards critical action to improve self-management [15, 17, 26–28]. The interventions facilitated participants to confront their illness--in some cases--for the first time [17, 23, 24, 26–28]. All studies noted participants developed trusting relationships with each other [15, 17, 23, 28], helped their fellow group members find meaning in stories [23, 24, 26–28], and problem-solved treatment and recovery issues [15, 24, 26, 28].

### Evaluating storytelling interventions

In some studies, the effects of storytelling interventions were assessed during and following the intervention sessions. The specific outcomes measured during the course of the storytelling sessions were psychosocial parameters, such as well-being [20, 25] mood, coping, stress, satisfaction with life, self-care [20], self-efficacy [12, 23, 24], self-motivation [27], and actions to improve self-care [27, 28]. Also physiological parameters that can affect disease status such as pain, blood pressure, lipid ratio, glycemic control [12, 17, 26] were assessed in several of the studies after completing the intervention sessions.

The remaining studies focused more on the feasibility [23] and processes [15, 17, 27, 28] of the intervention to better understand the storytelling design, implementation and participant experiences. These studies explored participants’ attendance at the intervention sessions as well as the acceptability of the intervention by the facilitators [12, 17, 23] and participants [12, 17]. For instance, participants were asked in the last session to reflect on their experience, what self-care management skills they learned in the story-sharing group, and offered feedback on ways to improve the intervention [12, 17, 23].

## Discussion

### Core principles of storytelling interventions

As revealed from the studies reviewed, storytelling is primarily used for the purposes of reflection on people’s experiences living with a chronic disease. Allowing people to tell stories about their life and illness experience is noted as therapeutic [8] as it can facilitate learning and coping with a chronic disease. This is done by fostering a venue where participants are actively engaged in directing the discussion and exchanging management, emotional, and social support to each other. Our review identified that the core element of a storytelling intervention is the process of unearthing meaning in the lived experience of illness. The act of telling a story can initiate the process of reflection and understanding [29] of oneself and the disease process. Through sharing of personal stories, individuals endeavors to give meaning to their illness and living with this illness, which could enhance a sense of personal control [30, 31]. As meaning unfolds, individuals may change the way they view their illness, which may yield changes in their approach to managing their condition and their health-related behaviours. With the clarity, understanding, and insight that meaning provides, a person may be better able to cope with the realities of their illness [31].

Overall, the themes that are discussed and emerge from the sessions appear to help participants understand their disease experience. Stories often start out as a single person’s narrative, but once elaborated by group members they become collectivized and shared experiences [15, 28]. Since stories materialize from social interchange, they can be described as social constructions [28] whereby fragments come together to create a whole. Feedback on stories was regularly given by group participants and the facilitator [28], as needed.

The anticipated impact of using storytelling in a group setting is the creation of a community among members where health education, health promotion, and support in self-management can be exchanged and ultimately self-management decisions can be made. In addition, this type of intervention creates an extended dialogue with other participants and with their healthcare provider about the illness and its management. Such dialogue can potentially act as a means to future problem-solving, attempts to change or improve self-management, and build relationships among participants and participants with their healthcare provider. The involvement of care providers as facilitators is also a learning opportunity to better understand, support and to care for their patients, in addition to fact checking information that is being shared, an element that may be absent in exclusive peer support groups.

The practice of sharing stories within a group is not just reserved for those whom storytelling is a valued cultural tradition. Storytelling interventions can potentially be conducted with most age groups, ethnicities (and held in different languages), socioeconomic status, or gender. It is possible that single-gendered interventions may make it easier for participants to more readily express their thoughts on disease issues relating to sexuality [28]. However, this is not to say that storytelling groups cannot be successfully made-up of participants from both genders. Some interventions also allow family and friends to sit-in on sessions to support participants and learn disease-specific information [15, 17, 26].

Combining the guiding principles of storytelling, such as informality, spontaneity, non-directional, equality, and community-building into one intervention, makes storytelling a unique self-management approach. All of these components are not typically offered together in one-one counseling or traditional self-management programs, and thus, these programs may fall short in facilitating patient empowerment. Storytelling’s core elements and guiding principles clearly demonstrate that it is carried out from the perspective of the patient using their needs as the focus.

#### Theoretical framework

Having storytelling interventions grounded in a theoretical or conceptual framework helps elucidate its active ingredients and the mechanisms underlying change in the outcomes of interest. It allows researchers to move past general insights to acknowledge and understand what underlying processes are causing the observed results; and how such results can be applied in every day practice [32], producing wider applicability of the interventions and significance to the study conclusions [32]. However, no single conceptual framework has specifically informed the storytelling approach as evidenced by the use of four different ones in the studies reviewed. Yet, there is a small, but growing literature on narrative theoretical frameworks for promoting health and shaping behaviour change that also incorporates storytelling as a narrative approach [33, 34]. In particular, Kreuter et al. [35] discuss a narrative framework specifically for the self-management of cancer that can be transferrable to other chronic diseases. These frameworks provide guidance for further development and application of storytelling as an intervention as well as future research aimed at evaluating its effectiveness.

#### Operationalization of the intervention - facilitators

A key factor to consider in implementing a storytelling intervention is the individual who will facilitate the sessions and the role responsibilities. In four of the reviewed articles, the facilitators were health professionals with experience in the disease-related field. These professionals were able to answer disease-specific questions and correct any faulty statements regarding the disease or its management, made by participants. Although resources are not necessarily always available to have health professionals facilitate such interventions, community members may be a more feasible option when working with specific cultural populations. A disadvantage here is that community members will need more training given the skills required to facilitate such an intervention as noted by Greenhalgh et al. [12]; these researchers found that some Bilingual Health Advocates lacked confidence in facilitating the sessions. Depending on the confidence, experience, and qualifications of the person leading the storytelling group, different levels of support would be required for each Bilingual Health Advocates [26]. Furthermore, there are high attrition rates of community health workers in health programmes [36] which would require continual training overtime.

Regardless of who facilitates the storytelling sessions, the facilitator must value the stories shared by participants in order to be effective [12]. They must also develop skills in implementing stories as a basis for reflection and discussion in the group. When facilitators ask questions such as, “What is happening here?” or “Why are things the way they are?” [28], it challenges participants to reflect and develop their own analysis of what is occurring in the group at that particular time. Facilitators should be focused on delicately drawing out stories from participants and maintaining group norms. Therefore, training is an important component in preparing facilitators to run storytelling sessions. In one study, the facilitator was an active participant in sharing stories as a way of role-modelling and encouraging trust [23]. By demonstrating themselves as storytellers, facilitators may be considered as part of the group without the typical hierarchy that exists within traditional context of provider-patient interactions; this may create an empowering atmosphere in which patients view themselves as knowledgeable through their lived experience of the disease. The facilitators ought to find a comfortable balance with their degree of involvement in the group; they must meet the needs of participants by becoming their peer or equal [28, 37], while still fulfiling the objectives of the storytelling intervention. The literature provides no clear indication of what type and how long facilitator training should last. Thus, it is unclear whether a formal curriculum is needed or if just a few training sessions will suffice. It will depend on whether the facilitators are health professionals or community peers, as health professional may already have experience in counselling and disease knowledge.

#### Operationalization of the intervention - atmosphere of sessions

The external and internal environment where storytelling takes place is key. The location for the intervention sessions should be familiar and easily accessible to participants in order to attract and maintain enrolment of group members over time. The actual space in which stories are shared has to make participants feel safe and relaxed [38]. The physical environment contributes to a sense of trust or security. Having participants lay the ground rules for how the sessions operate invites a sense of psychosocial ease and comfort [23]. This cooperative act of setting guidelines ensures an empathetic atmosphere where peer learning and support take place.

#### Operationalization of the intervention- session structure

There is no set rule on the optimal group size (i.e. number of participants in group sessions), the time length of a session, or the number of sessions recommended for storytelling interventions. The average number of sessions in the reviewed studies was 9 (excluding Greenhalgh’s [12] study). Caserta and Lund [39] recommended that 12 weeks be an optimal session number for people to develop relationships, allow for self-expression, and wade through issues together. However, Crogan et al., [24] reported 12 weeks was a lengthy commitment for participants, in contrast to Greenhalgh et al’s [40] finding that participants were disappointed to stop after 12 sessions. The reviewed studies did not offer insight into the most appropriate group size. Determining the ideal number of sessions and the desired group size may need to be based on participants’ preference and complexity of the self-management of the disease. 

#### Operationalization of the intervention- Participants’ roles

Once a program has been initiated, participants should be involved in selecting session topics so the intervention is participant-directed and responsive to their specific needs. One way to go about this is to have participants complete an informal survey to determine group session topics ahead of time. The advanced surveys can also assist in planning for a guest health professional to attend. After this occurs, participants can then be asked to prepare stories on the selected topics for the following session. This is another way participants have ownership over the sessions and feel empowered by the experience. This is in contrast to a traditional support group where content is spontaneous and conversations are random and untailored [24]. Otherwise support groups can provide opportunities to tell stories about illness and therapy.

#### Operationalization of the intervention- props to facilitate discussion

In complex medical conditions, the use of action-oriented activities such as props and other tools is important to provide an opportunity for kinesthetic (hands-on) learning. For example, learning how to correctly perform a task such as testing blood glucose levels can be confusing for people with diabetes mellitus. [41]. Having the opportunity to perform a behaviour or skill though storytelling can also assist participants in learning those skills [15]; this in turn, can increase individual self-management. Practicing certain skills enhances action orientated activities, as it is important for participants to see how things are done accurately to better manage their disease [26].

### Further recommendations

Strategies for storytelling could be in a verbal or written format or through the use of photographs. Greenhalgh et al. [12] stated that future intervention designs should couple storytelling with individual written goal-setting or care planning, since it has been linked with improved outcomes in diabetes peer support and education programs [42]. Such goal setting activities were a major component of the Sitvast [27] photo stories intervention [27]. In addition, depending on the literacy level of participants, other exercises such as homework assignments or journaling may help with individual goal-setting, self-reflection, and coping with illness [43]. Moreover, written assignments at home can serve as a reminder of self-management behaviours and may alleviate the stress that may arise from communicating with others face-to-face [44].

### Limitations and strengths

The use of storytelling as an approach for self-management interventions is novel and potentially useful for chronic disease self-management. Although a small number of papers was included in the review, representing eight independent interventions [17, 20, 25–28] of which two sets of articles [12, 15, 23, 24] describe the same intervention, findings identified the core elements of storytelling. The heterogeneity in diseases type, study design, topics discussed, and intervention implementation and evaluation, limited the ability to compare and validate storytelling’s efficacy on chronic disease self-management. In fact, many articles reported results of pilot, feasibility, or descriptive/qualitative studies. Further research is recommended to assess the efficacy of storytelling interventions for self-management in chronic illnesses.

## Conclusion

Given the challenges experienced by those living with chronic diseases, there is room for improvements in the support and care delivered through existing self-management programs. Storytelling may have the potential to be an effective approach or tool for chronic disease self-management interventions. Through this scoping review, we have identified guiding principles common across storytelling interventions that can direct further development and implementation of storytelling interventions in chronic disease management. Discovering meaning through self-reflection and critical action is at the core of storytelling interventions. It provides patients with a more active role in their health care, while allowing them to form strong bonds with peers who share similar disease-related experiences. The concept and implementation of storytelling can also provide healthcare professionals and educators with greater insight into their patients’ needs as well as an increased understanding of how patients manage and cope with their chronic illness.
